# Evolutionary Divergence of *Marinobacter* Strains in Cryopeg Brines as Revealed by Pangenomics

**DOI:** 10.3389/fmicb.2022.879116

**Published:** 2022-06-06

**Authors:** Zachary S. Cooper, Josephine Z. Rapp, Anna M. D. Shoemaker, Rika E. Anderson, Zhi-Ping Zhong, Jody W. Deming

**Affiliations:** ^1^School of Oceanography, University of Washington, Seattle, WA, United States; ^2^Astrobiology Program, University of Washington, Seattle, WA, United States; ^3^Department of Biochemistry, Microbiology and Bioinformatics, Université Laval, Québec, QC, Canada; ^4^Center for Northern Studies (CEN), Université Laval, Québec, QC, Canada; ^5^Institute of Integrative Biology and Systems (IBIS), Université Laval, Québec, QC, Canada; ^6^Department of Earth Sciences, Montana State University, Bozeman, MT, United States; ^7^Department of Biology, Carleton College, Northfield, MN, United States; ^8^Byrd Polar and Climate Research Center, Ohio State University, Columbus, OH, United States; ^9^Department of Microbiology, Ohio State University, Columbus, OH, United States; ^10^Center of Microbiome Science, Ohio State University, Columbus, OH, United States

**Keywords:** cryopeg, extremophile bacteria, evolution, ecology, pangenomics, oceanography, astrobiology

## Abstract

*Marinobacter* spp. are cosmopolitan in saline environments, displaying a diverse set of metabolisms that allow them to competitively occupy these environments, some of which can be extreme in both salinity and temperature. Here, we introduce a distinct cluster of *Marinobacter* genomes, composed of novel isolates and *in silico* assembled genomes obtained from subzero, hypersaline cryopeg brines, relic seawater-derived liquid habitats within permafrost sampled near Utqiaġvik, Alaska. Using these new genomes and 45 representative publicly available genomes of *Marinobacter* spp. from other settings, we assembled a pangenome to examine how the new extremophile members fit evolutionarily and ecologically, based on genetic potential and environmental source. This first genus-wide genomic analysis revealed that *Marinobacter* spp. in general encode metabolic pathways that are thermodynamically favored at low temperature, cover a broad range of organic compounds, and optimize protein usage, e.g., the Entner–Doudoroff pathway, the glyoxylate shunt, and amino acid metabolism. The new isolates contributed to a distinct clade of subzero brine-dwelling *Marinobacter* spp. that diverged genotypically and phylogenetically from all other *Marinobacter* members. The subzero brine clade displays genomic characteristics that may explain competitive adaptations to the extreme environments they inhabit, including more abundant membrane transport systems (e.g., for organic substrates, compatible solutes, and ions) and stress-induced transcriptional regulatory mechanisms (e.g., for cold and salt stress) than in the other *Marinobacter* clades. We also identified more abundant signatures of potential horizontal transfer of genes involved in transcription, the mobilome, and a variety of metabolite exchange systems, which led to considering the importance of this evolutionary mechanism in an extreme environment where adaptation *via* vertical evolution is physiologically rate limited. Assessing these new extremophile genomes in a pangenomic context has provided a unique view into the ecological and evolutionary history of the genus *Marinobacter*, particularly with regard to its remarkable diversity and its opportunism in extremely cold and saline environments.

## Introduction

Evolution and ecology are fundamentally entangled biological principles. The genomic variation that drives evolution can be introduced vertically, through stochastic mutations at the individual nucleotide level, and horizontally, *via* horizontal gene transfer (HGT) between organisms recognizable at the whole gene level, while the mutations or introductions that confer adaptation are selected by ecological conditions (Schloter et al., 2000; Brockhurst et al., 2019). Ubiquitous on Earth, including extreme environments, are the prokaryotes (Fenchel and Finlay, 2004). These morphologically simple organisms (single-celled, generally lacking internal compartmentalization) maximize metabolic efficiency by optimizing surface area to volume ratio (Harris and Theriot, 2018). Their lack of morphological variety has been a strength, rather than a limitation, allowing for extremely diverse genotypes encompassing enormous metabolic potential (Durot et al., 2009). Metabolic efficiency, diversity, and flexibility are essential components of global microbial ubiquity, but the mechanisms for acquiring the components that enable habitation of the many extreme environments on Earth are not well understood. Evidence for HGT has been obtained from extreme environments (e.g., Collins and Deming, 2013; Li et al., 2014; Raiger Iustman et al., 2015; Bosi et al., 2017; Moulana et al., 2020), but evaluations of the potential importance of this mechanism compared to its role in moderate environments are rare (e.g., Fuchsman et al., 2017).

Extreme environments are those with physical or chemical characteristics that are understood to limit the ability of life to function, such as temperatures near the freezing or boiling point of water (Harrison et al., 2013). The majority of inhabited environments on Earth are marine and cold, at temperatures below 5°C which slow the rate of metabolic reactions (Price and Sowers, 2004). Across the climate-threatened cryosphere (below 0°C) where water can remain liquid due to increased concentrations of salt ions and other impurities (Cox and Weeks, 1983), microbial life remains ubiquitous (Boetius et al., 2015). Though extreme environments present unique challenges to life, microorganisms can be highly abundant in them (e.g., >10^8^ ml^−1^ in subzero brines; Cooper et al., 2019), with niche competition continuing as an important selective pressure (Rapp et al., 2021).

**Figure 1:**
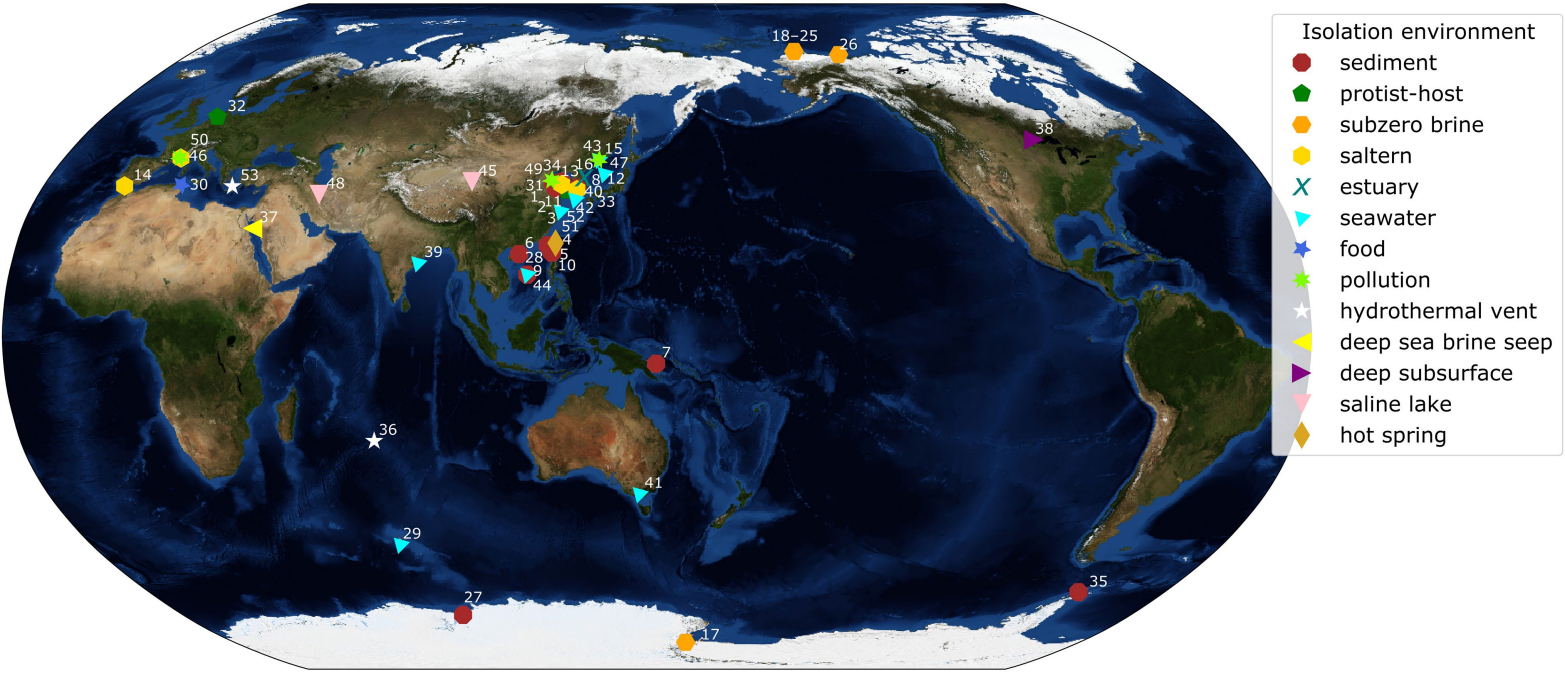
World map with isolation locations of each species of *Marinobacter* represented in the pangenome. Each symbol and color represents the isolation environment of the species or genome used here. Each point on the map is annotated with an ID number linking that point to its genome description in Supplementary Table 1. Annotations have been adjusted manually for readability and correspond to a point in the cluster if the area is too crowded. The background image used for the map is the Blue Marble: Next Generation Topography and Bathymetry image created by the NASA Earth Observatory.

A bacterial group globally distributed across the dominant cold and saline environments of Earth is the genus *Marinobacter* (Figure 1). A total of 45 complete genomes of species representative strains of *Marinobacter* were publicly available in the NCBI database at the start of this study (September 2020), while additional genomes of variable completion rates are available in this and other databases. This genus presents a potentially useful model for examining evolutionary mechanisms responsible for adaptation to extreme environments because its various members persist under conditions ranging from moderate to extreme, offering that comparison. Physiological studies have shown that this group of heterotrophic Gammaproteobacteria is metabolically diverse, capable of consuming a wide variety of organic compounds, including hydrocarbons, carbohydrates, and amino acids, and are broadly characterized as facultatively anaerobic (Gauthier et al., 1992; Green et al., 2006; Liu et al., 2012; Handley and Lloyd, 2013). The type species, *Marinobacter hydrocarbonoclasticus*, was isolated from hydrocarbon-polluted seawater near the mouth of an oil refinery (Gauthier et al., 1992). Other species have since been obtained from a variety of saline environments characterized by warm to extremely cold temperatures (precise *in situ* temperatures are not always reported in this literature). The species used in our study (listed in Supplementary Table 1, with geographic source indicated in Figure 1) were originally obtained from seawater (temperate and polar; Shivaji et al., 2005; Park et al., 2015; Vaidya et al., 2015), marine sediments (Montes et al., 2008; Han et al., 2017), hypersaline lakes (Bagheri et al., 2013; Zhong et al., 2015), deep-sea hydrothermal vent settings (Kaye et al., 2011; Wang et al., 2012), deep-sea temperate and cold brine seeps (Antunes et al., 2007; Sun et al., 2020), deep subsurface saline deposits (Bonis and Gralnick, 2015), and the polar subzero habitats of sea ice (Canadian Basin; Zhang et al., 2008), hypersaline springs in permafrost (Axel Hieberg Island, Canadian High Arctic; Niederberger et al., 2010), Arctic sea ice (Zhang et al., 2008), and Antarctic brines from Lake Vida (Kuhn et al., 2014) and Blood Falls (Chua et al., 2018). Members of this genus thus exploit an opportunistic lifestyle that enables them to persist and even thrive in saline and frequently cold environments, including those under the most extreme conditions in polar regions.

Recently, we identified *Marinobacter* as the dominant genus inhabiting cryopeg brines found near Utqiaġvik, Alaska (Cooper et al., 2019). Cryopegs are liquid-saturated sediment layers within permafrost that remain unfrozen at subzero temperatures due to their salt content (Gilichinsky et al., 2003, 2005). The cryopeg system we sampled is approximately 2 km from the modern Arctic coastline and most likely derived from marine sediment that was exposed to subzero temperatures and incorporated into permafrost at least 40 ka BP based on radiocarbon dating (Meyer et al., 2010a,b; Iwahana et al., 2021). For the last 40 ka, the cryopeg brines have been isolated from external influences, with observations indicating relatively stable subzero temperatures (−8 to −6°C) and hypersalinities (109–140 ppt; Cooper et al., 2019; Iwahana et al., 2021). The microbial communities inhabiting these subzero brines are dense (approximately 10^8^ cells ml^−1^), with *Marinobacter* accounting for, on average, 49% of the prokaryotic community, based on its relative abundance generated by 16S rRNA gene amplicon sequencing (Cooper et al., 2019). Though not dominant in the sea-ice brines we sampled, previous work showed that *Marinobacter* can be dominant in Arctic sea ice, composing approximately 22% of the bacteria present (Brinkmeyer et al., 2003) as well as in the brines of Antarctic ice-covered Lake Vida (Murray et al., 2012; Kuhn et al., 2014). Although the *Marinobacter* spp. previously isolated from other subzero brines in Arctic and Antarctic settings were not reported as dominant *in situ* (Zhang et al., 2008; Niederberger et al., 2010; Chua et al., 2018), the common occurrence of *Marinobacter* spp. in subzero hypersaline environments in Earth’s polar regions raises questions about the evolutionary history and adaptations of this genus that give it a competitive advantage under such extreme conditions.

Here, we use pangenomics (Tettelin et al., 2005, 2008; Delmont and Eren, 2018; Brockhurst et al., 2019) to examine the genomic potential of the genus *Marinobacter*, with a particular focus on newly obtained members from cryopeg brines. Pangenomics is a method of genomic analysis that compares the gene content of a set of closely related genomes, generally at the genus or species level and often with a focus on the frequency of gene occurrence among genomes. It allows for exploration of the characteristics that are common within a group and for discovery of characteristics that may distinguish a subset of the group. Pangenomics has been applied to help understand the ecology and evolution of many microbial taxa, such as the globally distributed bacterial genus *Prochlorococcus*, a photosynthesizing genus cosmopolitan to ocean surface waters (Biller et al., 2014 Delmont and Eren, 2018). It has also been applied to *Sulfurovum*, a mesophilic chemolithoautotrophic genus ubiquitous in hydrothermal vent environments, to investigate the influences of biogeography and environmental conditions on genomic differentiation (Moulana et al., 2020). Motivating this study was the successful acquisition of new genomes of *Marinobacter* from the Alaskan cryopeg brines we have been studying (Cooper et al., 2019; Iwahana et al., 2021; Rapp et al., 2021) using both classical cultivation methods and sample recovery of metagenome-assembled genomes (MAGs), in the latter case using short-read (Illumina) and long-read (Nanopore) assembly approaches to maximize their quality. These genomes add novelty to the known diversity of this genus and allow a new focus on the cryosphere. Using the pangenome generated from these cryopeg genomes and the publicly available representative genomes of other reported *Marinobacter* species, we explored the evolutionary history, functional capabilities, and pangenomic diversity of this genus, including the potential role of horizontal gene transfer in adapting to the extreme conditions of subzero, hypersaline environments.

## Materials and Methods

### Isolation of *Marinobacter* Strains From Cryopeg Brines

Cryopeg brines were collected near Utqiaġvik, Alaska, in May 2018 as previously described (Cooper et al., 2019). Samples were frozen at −20°C in the field laboratory and returned to the University of Washington (Seattle, WA, United States). Cryopeg brine, labeled CBIW_18 (as described by Cooper et al., 2019), was used as the inoculum for bacterial isolation after thawing from −20°C to −1°C over several days. The brine was diluted serially to a final dilution of 10^−9^ in a variety of defined media, based on ONR7a, each containing a single organic substrate (L-alanine, L-leucine, Na-acetate, kerosene, or 1-butanol) at 10 mM concentrations. ONR7a is a minimal medium used to isolate marine bacteria that degrade hydrocarbons or other individual organic substrates (Dyksterhouse et al., 1995). ONR7a is composed of three salt solutions that are combined after preparation: (1) 22.79 g NaCl, 3.98 g Na_2_SO_4_, 0.72 g KCl, 83 mg NaBr, 31 mg NaHCO_3_, 27 mg H_3_BO_3_, 2.6 mg NaF, 0.27 g NH_4_Cl, 89 mg Na_2_HPO_4_ x 7H_2_O, and 1.3 g TAPSO in 500 ml H_2_O with pH adjusted to 7.6 with NaOH; (2) 11.18 g MgCl_2_ x 6H_2_O, 1.46 g CaCl_2_ x 2H_2_O, and 24 mg SrCl_2_ x 6H_2_O, in 450 ml H_2_O; and (3) 2.0 mg FeCl_2_ x 4H_2_O, in 50 ml H_2_O. All of the sample dilutions were incubated at 2°C for 66 days, when growth was observed in each 10^−7^ dilution but not in the 10^−8^ or 10^−9^ dilutions. From each of the 10^−7^ dilution cultures, 100 μl was transferred to solid plate media consisting of 1.2% agar and half-organic-strength Difco Marine Broth 2216 (Fisher, Waltham, MA, United States), amended to full seawater strength with artificial seawater composed of 24 g NaCl, 0.7 g KCl, 5.3 g MgCl_2_ x 6H_2_O, MgSO_4_ x 7H_2_O, and 1.3 g TAPSO in 1 L H_2_O with pH adjusted to 7.5. Colonies were picked from agar plates and streaked three times until a final colony was transferred to liquid Marine Broth for growth.

Screening of genus-level taxonomic identity of isolates (two from each ONR7a + organic substrate medium) was conducted using Sanger sequencing of the 16S rRNA gene. For 16S rRNA gene sequencing, cell pellets from liquid cultures were sent to GENEWIZ, Inc. (South Plainfield, NJ, United States) where DNA was extracted and sequenced. Isolates confirmed as *Marinobacter* came from media containing alanine (*n* = 2), acetate (*n* = 1), or kerosene (*n* = 1) as the sole organic carbon source. The two isolates from the alanine medium were designated M1C and M2C, and the isolates from kerosene and acetate media were designated M3C and M4C, respectively.

### Genome Sequencing

Pelleted cultures of the four selected isolates were extracted and sequenced at the Microbial Genome Sequencing Center (MiGS; Pittsburgh, PA, United States). DNA extraction, library preparation, genome sequencing, quality control, and assembly were performed at MiGS. Briefly, the genomes were sequenced using both Illumina short-read and Oxford Nanopore Technologies (ONT) long-read sequencing technologies. Quality control of the raw Illumina and ONT reads was performed using bcl2fastq (Illumina) and Porechop (Wick et al., 2017a), respectively. Hybrid assembly of the reads was conducted using Unicycler (Wick et al., 2017b). After receiving the assembled genomes, we checked genome completeness using CheckM v1.1.2 (Parks et al., 2015) and assigned the taxonomy of each genome using GTDB-tk v1.1.1 (Parks et al., 2018). Gene calling was performed in anvi’o v6.1 (Eren et al., 2015) using Prodigal v2.6.3 (Hyatt et al., 2010) to identify open reading frames (ORFs). Each ORF was annotated with National Center for Biotechnology Information (NCBI) Clusters of Orthologous Genes (COG) annotations (Tatusov et al., 1997; Galperin et al., 2019) which were assigned using DIAMOND v0.9.22.123 (Buchfink et al., 2014). These genome sequences have been deposited in GenBank and are available under accession numbers CP092283–CP092289.

### Metagenome Sequencing, Assembly, and Binning

DNA from cryopeg brine samples (25–500 ml; *n* = 5), concentrated onto 0.22 μm Sterivex filters in the field and kept frozen at −80°C until processing, was extracted using the DNeasy PowerSoil kit (QIAGEN, Germantown, MD, United States) as previously described (Cooper et al., 2019). Samples collected for DNA extraction for this study are identical to samples CBIW_17, CBIW_18, CBIA_18, and CB1_18 described by Cooper et al. (2019). Sample names for CBIA_18 and CB1_18 were simplified to CBIA and CB1 because, unlike CBIW_17 and _18, they were sampled only in 1 year (2018). Metagenomic sequencing was conducted using both Illumina short (2 × 150 bp) and ONT long reads. The Illumina sequencing library was prepared, sequenced, and analyzed at the DOE Joint Genome Institute as previously described (Rapp et al., 2021), and the ONT sequencing library was prepared, sequenced, and quality-controlled following the VirION2 pipeline (Zablocki et al., 2021). Long reads were first assembled using Flye v2.5 (Kolmogorov et al., 2019), and short reads were subsequently used for error correction using Pilon v1.23 (Walker et al., 2014). Each metagenome was assembled from a single sample of cryopeg brine, allowing for recovery of MAGs from discrete samples. The raw Illumina metagenomic reads were produced previously and have been described in detail by Rapp et al. (2021); they are available through IMG/M under accession numbers 3300031836 (CBIW17), 3300032129 (CBIW18), 3300032135 (CBIA), and 3300034171 (CB1). The long-read metagenomic reads used to generate the polished assemblies produced here are available in the Sequence Read Archive under accession numbers SRR17952620, SRR17952621, SRR17952622, and SRR17952623.

Assembled metagenomic contigs were processed using anvi’o v6.1 (Eren et al., 2015). Gene calling for the metagenomes was performed in anvi’o as described above for the whole isolate genomes. Bacterial and archaeal single copy universal marker genes (Lee, 2019) were identified using HMMER v3.3 (Eddy, 2011). Automated binning was conducted using MetaBAT 2.0 v2.15 (Kang et al., 2019), MaxBin2 v2.2.7 (Wu et al., 2016), and Concoct v1.1.0 (Alneberg et al., 2014). The best bins from each tool were selected automatically using DAS Tool v1.1.2 (Sieber et al., 2018). After binning, the bins were refined manually in anvi’o using coverage, gene clustering, and GC content as guides (https://merenlab.org/data/refining-mags/, accessed 19 May 2020). After binning, each MAG was assigned taxonomy using GTDB-tk v1.1.1 (Parks et al., 2018), and MAG completeness was estimated using CheckM v1.1.2 (Parks et al., 2015). The MAGs in this study are designated CBIW17, CBIW18, CBIA, and CB1 referencing the corresponding original sample name. The MAG sequences have been deposited in DDBJ/ENA/GenBank where they are available under accession numbers JAKRQQ000000000, JAKRQR000000000, JAKRQS000000000, and JAKRQT000000000. The versions described in this paper are versions JAKRQQ010000000, JAKRQR010000000, JAKRQS010000000, and JAKRQT010000000.

### Pangenomic Analysis

Along with the four MAGs (CB1, CBIA, CBIW17, and CBIW18) and four isolate genomes (M1C, M2C, M3C, and M4C) we obtained in this study, 45 representative genomes of *Marinobacter* spp. were acquired from the NCBI Assembly database (on 16 September 2020) as genomic FASTA files. References and accession numbers for each genome are listed in Supplementary Table 1. We followed the standard pangenomic workflow in anvi’o v6.1 (Delmont and Eren, 2018; Moulana et al., 2020) to build and analyze the *Marinobacter* pangenome from 53 total genomes. We created a contig database for each isolate and representative genome using the command “anvi-script-fasta-to-contigs-db”; this script performs gene calling as described in the sections above. Each genome was annotated with NCBI COGs as described above. Original metagenomic contig databases were used for each MAG. We combined all genomic data by creating a genome storage database using the command “anvi-gen-genomes-storage” and specifying paths to internally stored contig databases for the MAGs (using data already processed at the metagenome level) and external paths to the rest of the *Marinobacter* contig databases. Then, the *Marinobacter* pangenome was assembled from the genome storage database using the command “anvi-pan-genome” with the “--use-ncbi-blast” flag which directs the program to use BLASTp (Altschul et al., 1990) for calculating amino acid similarities between related gene calls. Using the default settings, the “anvi-pan-genome” program employed a Markov Cluster Algorithm (MCL; van Dongen and Abreu-Goodger, 2012) which clustered highly similar gene calls using the BLASTp search results to allow for assessment of gene overlap between individual genomes. The pangenome database produced contains information on gene cluster presence/absence and abundance for each genome and gene cluster similarity across all genomes.

### Phylogenetic Inference

To assess the phylogenetic relationships among the members of the *Marinobacter* genus, we constructed phylogenetic trees using three approaches. In the first, we calculated average nucleotide identity (ANI) across the *Marinobacter* pangenome using the anvi’o program “anvi-compute-genome-similarity” which employed the program pyANI v0.2.10 (Pritchard et al., 2016) using the ANIb option. The ANIb option uses BLASTN+ to align 1,020 nt fragments of input data and calculate similarity. The program produced a matrix of ANI values for shared genomic regions, and anvi’o produced an hierarchically clustered phylogenetic tree using Euclidean distance and Ward clustering methods by default, which we call the “ANI” phylogenetic tree.

To construct the two additional phylogenetic trees, which we call the “core” and “rooted universal” trees, we used, respectively, a set of 108 single copy genes found in all genomes from the *Marinobacter* pangenome (the “core” genes) and a suite of universal bacterial single copy marker genes (Lee, 2019) found in all genomes across the pangenome, with the model psychrophilic bacterium *Colwellia psychrerythraea* 34H (Methé et al., 2005) as an outgroup. To generate the core phylogenetic tree, genes were selected from the *Marinobacter* pangenome using the program “anvi-get-sequences-for-gene-clusters” filtering for gene clusters that have a maximum functional homogeneity of 0.90 and a minimum geometric homogeneity of 0.95 that occur only once in each genome and that occur in a minimum of 53 of the genomes. The “--concatenate-gene-clusters” flag was used to specify the output format appropriate for phylogenetic tree generation. To generate the rooted universal tree, the genome for *C. psychrerythraea* 34H was first downloaded from NCBI, then processed similarly to all other genomes in the *Marinobacter* pangenome. HMM hits from all *Marinobacter* genomes and the *Colwellia* genome were aggregated using the program “anvi-get-sequences-for-hmm-hits” and specifying “--hmm-source Bacteria_71” and that the genes occur in at least 54 of the input genomes. The flags “--return-best-hit,” “--get-aa-sequences,” and “--concatenate” were used to get the best possible protein sequences for phylogenetic tree construction. Genes used in the construction of both trees are listed in Supplementary Data Sheet. Both the core and rooted universal phylogenetic trees were constructed using FastTree v2.1.10 (Price et al., 2010), which uses the Jones–Taylor–Thorton evolutionary model and Shimodaira–Hasegawa test (1,000 resamplings) to optimize tree topology. All trees were visualized using FigTree v1.4.4 (Rambaut, 2009). Clades of *Marinobacter* were defined operationally based on the branching patterns of the core phylogenetic tree.

### Metabolic Inference

Metabolic pathway completeness was estimated for each genome in the pangenome using KofamScan v1.3.0 (Aramaki et al., 2020) to assign Kyoto Encyclopedia of Genes and Genomes (KEGG) Orthologs (KOs) to each gene. Protein sequences for each gene were recovered for each genome using the anvi’o program “anvi-get-sequences-for-gene-calls” with the “--get-aa-sequences” flag. KofamScan was run for each genome using KO profiles, and lists were downloaded on 19 January 2021. KO annotation results were aggregated and analyzed using KEGG-Decoder v1.2.2 which assesses percent completeness of metabolic pathways defined by KEGG (Graham et al., 2018). A heatmap of the results was produced using a custom python v3.7.8 (McKinney, 2010; Reback et al., 2020; Waskom, 2021) script available at https://github.com/zscooper/marinobacter_pangenomics.

Functional enrichment based on COG annotations for each clade was assessed using the “anvi-get-enriched-functions-per-pan-group” program in anvi’o (Eren et al., 2015). The program uses a generalized linear model and applies the logit link function to compute an enrichment score and value of *p* for each gene cluster in the pangenome. The program then applies a false detection rate correction to the value of *p* to obtain a *q*-value for inference of significance (Storey and Tibshirani, 2003).

### Prediction of Horizontal Gene Transfer

We identified putative horizontally transferred genes using HGTector v2.0b3 (Zhu et al., 2014). Briefly, this program searches each gene in a genome, compares it to a taxonomy-informed database, and assesses the distribution of best hits to closely and more distantly phylogenetically related taxa to determine whether a gene is likely the result of vertical inheritance or HGT. To accomplish this task, HGTector uses a database consisting of NCBI RefSeq protein sequences; we downloaded the pre-built default database of reference and representative microbial genomes (dated 21 October 2019) found on the program manual web site. After downloading and decompressing it, the database was compiled using DIAMOND v2.0.6.144 (Buchfink et al., 2014). As input for HGTector2, we provided gene calls from each genome in the *Marinobacter* pangenome, the same as used for metabolic inference above. The “hgtector search” function was run on each genome individually using DIAMOND to score each gene against the database. After searching for best gene matches, “hgtector analyze” was run using a folder of all search results as input. For the analysis, the “--self-tax” was set to “2742” (NCBI taxon ID for the genus *Marinobacter*), the “--self-rank” was set to “genus,” the “--self-low” flag was set to “low,” the “--close-tax” was set to “72275” (NCBI taxon ID for the family Alteromonadaceae), and the “--bandwidth” method was set to “grid.” These settings were implemented in order to specify that best gene hits to *Marinobacter* or within Alteromonadaceae are not counted as horizontally transferred and to carefully identify cutoff for HGT determination. HGTector2 output was combined with NCBI COG (Galperin et al., 2019) annotations for each gene call assigned during pangenome assembly. Genes that were not annotated with COGs but scored as putative horizontally transferred genes were counted as “None.” The output data were analyzed and visualized using custom python v3.7.8 (McKinney, 2010; Reback et al., 2020; Waskom, 2021) scripts available at https://github.com/zscooper/marinobacter_pangenomics.

## Results

### Genome Characteristics of the Cryopeg Isolates of *Marinobacter*

Following the successful cultivation and genome sequencing of new isolates of *Marinobacter* and the recovery of high-quality MAGs from our subzero cryopeg brines, we first examined the basic characteristics of these new genomes for comparative purposes (Supplementary Table 2). For three of the new isolates, M1C, M2C, and M4C, each genome assembled into a single circular contig with lengths ranging from 4,745,310 to 4,747,818 bp, while M3C assembled into one large circular contig (4,762,857 bp) and three smaller circular contigs that are likely plasmid sequences (1,983–210,472 bp). All four of these genomes were scored as 100% complete according to lineage-specific single-copy marker gene detection (Parks et al., 2015). The four MAGs generated from the cryopeg brines (CBIW17, CBIW18, CBIA, and CB1) ranged in length from 3,085,923 to 4,242,133 bp and were 79%–92% complete. GC content ranged from 54.2%–54.4% for all eight of these novel genomes. The isolate genomes encoded 4,395 to 4,687 genes. Across the *Marinobacter* pangenome, the mean (±SD) genome length was 4,112,097 ± 444,972 bp, GC content was 56.9 ± 2.2%, and number of genes was 3,826 ± 418. Genome statistics for all genomes included in the pangenome are provided in Supplementary Table 2.

### Phylogeny and Genomic Similarity of the Genus

**Figure 2:**
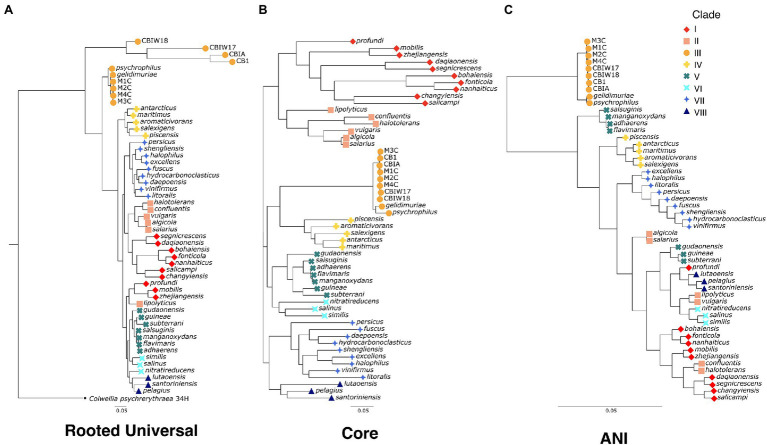
Phylogenetic trees for the genus *Marinobacter* **(A)** “rooted universal,” **(B)** “core,” and **(C)** “ANI”-based phylogenetic trees for the eight new genomes from cryopegs and 45 other representatives of *Marinobacter* species. The rooted universal tree **(A)** is constructed from a set of universal single copy bacterial marker genes (*n* = 69) and includes *Colwellia psychrerythraea* 34H as an outgroup. The core tree **(B)** is constructed from a set of genes found in single copies in each genome that have ≥95% geometric homogeneity and ≤90% functional homogeneity across the pangenome (*n* = 108). The ANI tree **(C)** represents the relatedness of each genome based on percentage identity of shared genomic content. The leaves of each tree are coded by color and shape according to the clade to which they belong.

To evaluate the evolutionary relatedness of the newly generated genomes of *Marinobacter* to other members of the pangenome presented here, we used three methods of phylogenetic inference. In one method, we constructed a rooted universal tree, using a subset of the universal bacterial single copy marker genes (*n* = 69; Lee, 2019) found in every genome in the pangenome (Figure 2A). In another, each genome was assigned to a clade based on branching patterns of the core phylogenetic tree, based on single copy core genes (Figure 2B). This unrooted core tree, consisting of the 108 genes found in gene clusters that had a geometric homogeneity score of at least 0.95 and a functional homogeneity of less than 0.90, ensures a high level of both sequence alignment and functional diversity. For comparison, we also constructed a maximum likelihood phylogenetic tree, based on ANI values using Euclidean distance and Ward clustering (Figure 2C). The rooted universal phylogenetic tree (Figure 2A) indicates that Clade III, which consists of the novel *Marinobacter* genomes from cryopeg brines and the genomes from *Marinobacter* spp. previously isolated from subzero brines in the Arctic (Zhang et al., 2008) and Antarctic (Chua et al., 2018), branches before the rest of the genus. Clade III is paraphyletic in the rooted universal tree and monophyletic in both core (Figure 2B) and ANI (Figure 2C) trees. Clades IV, VI, VII, and VIII also consistently cluster monophyletically, while clades I, II, and V cluster inconsistently across trees. Clades I and III correspond with the environment of isolation of each species representative, while environmental distinction is not as clear for other clades (Supplementary Table 2).

**Figure 3:**
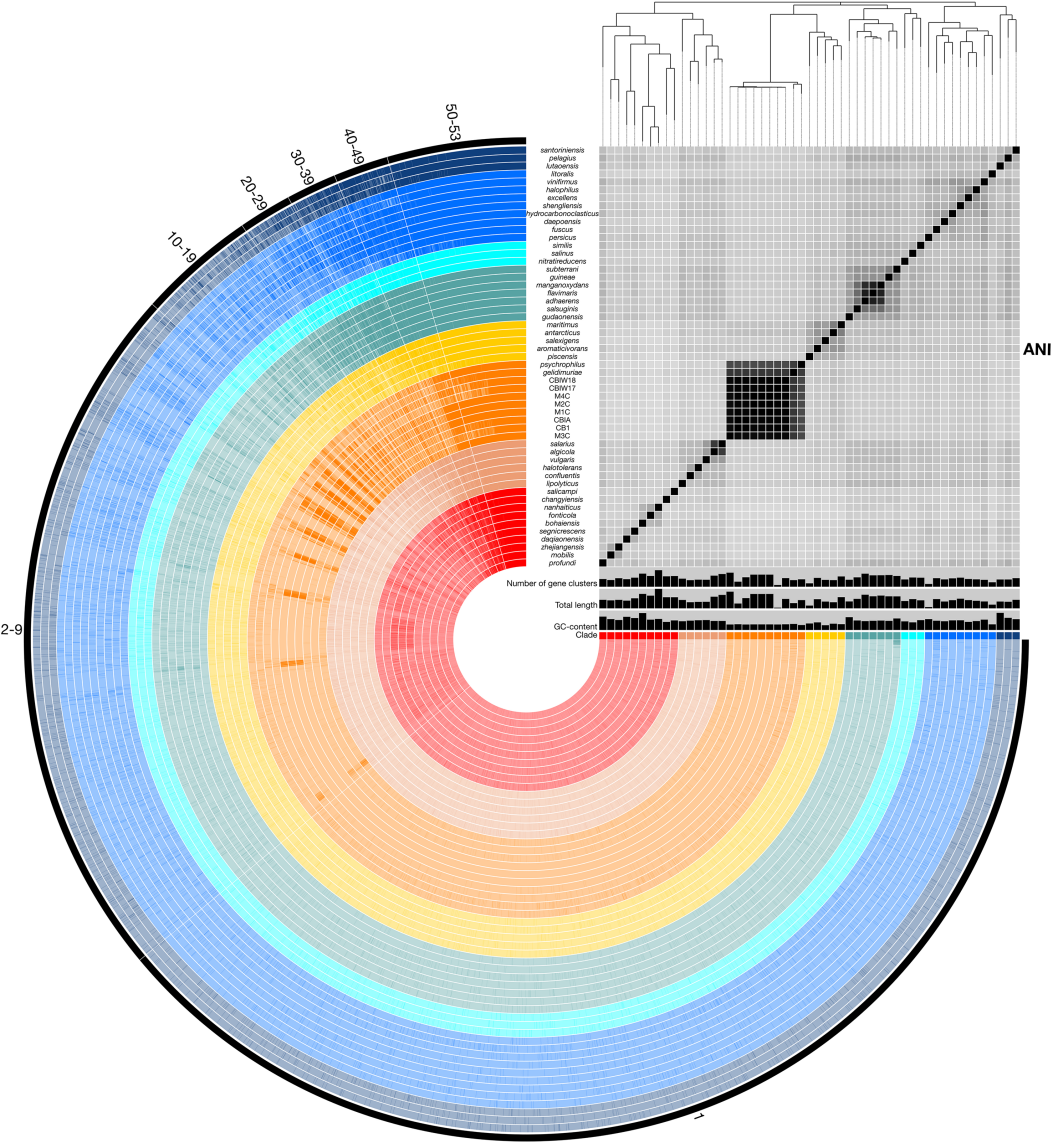
Representation of the *Marinobacter* pangenome with ANI heatmap. Interior circles are colored according to the clade to which the species belongs. Clades are ordered from I (red) to VIII (dark blue) from the innermost to outermost rings. Brighter lines in these interior circles of the pangenome represent gene cluster presence in each genome. Numbered exterior bars indicate the number of genomes containing gene clusters in each region of the pangenome. The bar graphs display GC content (50%–65%), total genome length (3,000,000–5,358,909 bp), and number of gene clusters (2,500–5,000) per genome. The heatmap represents ANI values between shared regions of each genome (70%–100%). The phylogenetic tree above the heatmap displays the core-gene phylogeny of the pangenome and serves as the basis for clade designations.

To assess relatedness of *Marinobacter* members, ANI was calculated between each genome in the pangenome based on sequence similarity of shared gene content. ANI and alignment coverage values for the pangenome are provided in Supplementary Data Sheet. The mean (±SD), median, minimum, and maximum percent identity of shared genes across the pangenome were 77.1 (±6.1)%, 75.2%, 72.7%, and 100%, respectively. Figure 3 displays a heatmap of ANI across the pangenome. The novel isolate genomes and MAGs in Clade III have a mean ANI of 99.5 (±0.47)% to each other, 90.7 (±1.2)% to *Marinobacter psychrophilus*, and 93.1 (±1.6)% to *Marinobacter gelidimuriae*. Clade III has a mean ANI of 74.7 (±0.81)% to all other genomes. Alignment coverage is low and highly variable across the pangenome with a mean of 44.0 (±16.4)%, median of 39.5%, minimum of 19.1%, and maximum of 100%. Histograms of the distribution of percent identity and alignment coverage scores are provided in Supplementary Figure S1. In addition to ANI, we found that Clade III genomes also have a significantly lower GC content than all other clades (54.3% vs. 57.6%; *p* = 2.7 × 10^−6^).

Ribosomal genes recovered from the genomes, each isolated from separate field samples, indicate that the novel cryopeg isolates all share a 100% identical 16S rRNA gene. The closest relatives by 16S rRNA gene similarity to these isolates are *M. gelidimuriae* and *M. psychrophilus* with 99.3% and 99.1% similarity, respectively. The remaining species of *Marinobacter* have a 16S rRNA gene similarity to the isolates of ≤97.2%. Genomic alignment coverage is generally low between species of *Marinobacter* (Supplementary Figure S1), and the cryopeg genomes have an alignment coverage of 61.4% to *M. gelidimuriae* and 71.3% to *M. psychrophilus*.

### Scope and Distribution of the *Marinobacter* Pangenome

Pangenomic analysis allows for an evaluation of conserved genes within a group of closely related genomes. We used this analysis to explore the diversity of genome-encoded pathways available to this genus and to identify the genomic features that distinguish the newly produced genomes from the rest of the genus. The *Marinobacter* pangenome constructed here consists of all of the genes found across 53 genomes: 45 species representatives for the genus, four newly sequenced genomes from isolates of *Marinobacter* from cryopeg brines, and 4 MAGs assembled from metagenomes sequenced from cryopeg brines. Isolation environments for these *Marinobacter* spp. are distributed across the globe, primarily in marine or terrestrial hypersaline settings (Figure 1). As the pangenome was constructed, genes from each genome were clustered to identify shared content between genomes. The pangenome contained a total of 24,580 gene clusters containing 202,751 total genes across all 53 genomes. Here, the distribution of gene content in the pangenome is described by the number of gene clusters shared by a range of a number of genomes, which includes a first set of all or almost all of the genomes (50–53) then divided further into decreasing sets of 10 until reaching singleton gene clusters (appearing in a single genome; Figure 3). Across the first comprehensive set of *Marinobacter* genomes, 1,465 gene clusters were shared, approximating the “core” of the pangenome. With decreasing numbers of genomes, the number of shared gene clusters was 579 found in 40–49 genomes, 532 found in 30–39 genomes, 561 found in 20–29 genomes, 1,243 found in 10–19 genomes, and 7,428 found in 2–9 genomes. The largest number of gene clusters, 12,772, each appeared in only a single genome (singletons). Gene cluster annotations and genome contributions are provided in Supplementary Data Sheet. Genes involved in translation, ribosomal structure, and biogenesis (COG category J) are the most abundant category, comprising 13.0% of the gene clusters in the core range of the pangenome. Category J gene clusters diminish in abundance consistently into the regions of the pangenome where fewer genomes share genes and comprise only 0.7% of the singleton gene clusters. Mobilome genes (COG category X) are not abundant in the core range but increase in abundance in the regions of the pangenome where fewer genomes share genes, making up 0.07% of the core and 2.0% of all singleton gene clusters. The number of gene clusters without functional annotations increases steadily from 4.8% of the core range to 56.9% of the singleton gene clusters (Supplementary Data Sheet).

### Metabolic Potential of *Marinobacter* spp.

**Figure 4:**
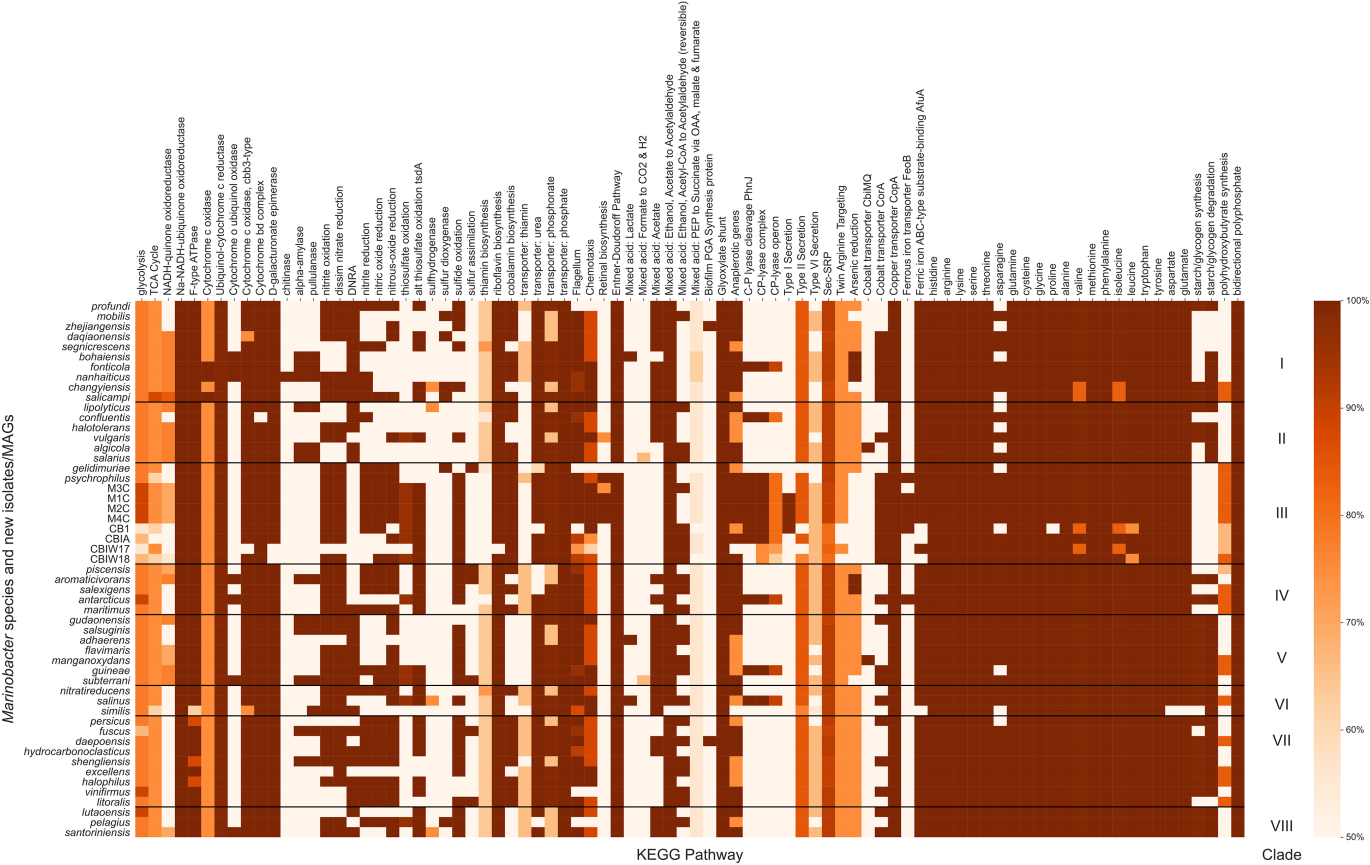
Heatmap of the completeness level of Kyoto Encyclopedia of Genes and Genomes (KEGG) metabolic pathways identified by KEGG Decoder for *Marinobacter* genomes. All pathways plotted were at least 50% complete in at least one genome. The colormap ranges from 50% (lightest orange) to 100% (darkest orange). Each row represents a single *Marinobacter* genome, and each column represents a metabolic pathway. Black horizontal lines separate the heatmap into clades with clade numbers listed between the heatmap and the scale bar.

*Marinobacter* spp. are known from physiological and environmental studies to perform a broad suite of metabolic functions (Handley and Lloyd, 2013), but a complete survey of metabolic potential encoded in the genomes of *Marinobacter* has not been published. We performed this survey to assess the variety of metabolisms that *Marinobacter* spp. encode and to identify metabolisms likely relevant to inhabiting cryopeg brines. Metabolic pathway completeness was assessed by assigning KOs to each gene in each genome and then using KEGG Decoder (Graham et al., 2018) to assess the proportion of KOs required for a pathway to be present. Values for KEGG pathway completeness for all genomes in the *Marinobacter* pangenome are provided in Supplementary Data Sheet; a subset, where at least one genome has 50% of a complete pathway, is displayed in Figure 4. Here, we highlight metabolic pathways that appear as important to the entire genus of *Marinobacter* and those that represent features unique to Clade III. Nearly all *Marinobacter* genomes (except only *M. gelidimuriae*) encode the genes required for the Entner–Doudoroff pathway, while none of the genomes encodes a complete glycolysis pathway. All *Marinobacter* genomes encode for the glyoxylate shunt. Almost all *Marinobacter* spp. encode metabolisms for each of the 20 standard amino acids. An exception is the metabolism of asparagine, which is nevertheless encoded by 41 of the 53 genomes in the pangenome.

Clade III genomes (with the exception of the MAGs which are not complete genomes) encode complete pathways (as defined by Graham et al., 2018) for nitrite oxidation and reduction, dissimilatory nitrate reduction, nitric oxide reduction, and nitrous oxide reduction, each of which is common among other *Marinobacter* species. Pathways for dissimilatory nitrate reduction to ammonium are not present in Clade III genomes, and no nitrogen fixation pathways are present in any *Marinobacter* genome. The potential to oxidize thiosulfate appears in the cryopeg genomes, while the pathways involved in this process are not present in other Clade III genomes (*M. psychrophilus* and *M. gelidimuriae*) and are sporadically present in other clades of *Marinobacter* (only complete for five other species). Whereas all *Marinobacter* genomes encode for the copper transporter CopA, and nearly all encode for the ATP-binding cassette (ABC)-type substrate-binding protein AfuA for ferric iron (the exceptions are three of the cryopeg MAGs and *M. similis*), only a subset of genomes encode for the cobalt transporter CorA (including Clade III, except for *M. gelidimuriae*) and several encode for the ferrous iron transporter FeoB (including M1C, M2C, M4C, and *M. psychrophilus*). Cryopeg isolates M1C, M2C, and M4C and MAG CB1 each encode a complete pathway for Type I secretion systems, while this system is mostly absent in the other *Marinobacter* genomes. Type II and Type VI secretion systems are nearly complete for the majority of *Marinobacter* genomes, while Type III and Type IV secretion systems appear to be absent from the pangenome. Clade III genomes M1C, M2C, M4C and *M. psychrophilus* encode complete pathways for retinal biosynthesis which involves carotenoid and rhodopsin biosynthesis; this pathway is less complete in other Clade III genomes and nearly absent in most other *Marinobacter* genomes. Clade III genomes (except for some MAGs and *M. gelidimuriae*) each encode for a nearly complete carbon-phosphorous (C-P) lyase system. Clade III genomes also encode a nearly complete pathway for polyhydroxybutyrate synthesis, which has variable levels of completion for all other genomes in the pangenome.

### Enrichment of Transporters and Transposases in Subzero Brine *Marinobacter* spp.

In a continuation of our effort to identify distinguishing features of the newly acquired *Marinobacter* genomes, we conducted an analysis using anvi’o to detect which gene clusters, annotated with COG functions, were enriched in each clade relative to their presence in other clades. This analysis revealed a variety of genes that were enriched solely in Clade III, the top 17 of which have an adjusted *q*-value <0.01. The top three functions enriched in Clade III are the permease, periplasmic, and ATPase components of the ABC-type uncharacterized transport system YnjBCD (COG4134, COG4135, and COG4136). Other functions enriched solely in Clade III include stress response protein SCP2 (COG2310), bacteriorhodopsin (COG5524), tellurite resistance protein TerB (COG3793), the 4Fe-4S-binding domain of the Fe-S cluster biogenesis protein NfuA (COG0694), the gamma subunit of sarcosine oxidase (COG4583), recombination-promoting DNA endonuclease RpnC/YadD (COG5464; previously annotated as a predicted transposase), and a putative component of the toxin-antitoxin plasmid stabilization module (COG3657). The complete results of this functional enrichment analysis are provided in Supplementary Data Sheet.

### Horizontal Gene Transfer

**Figure 5:**
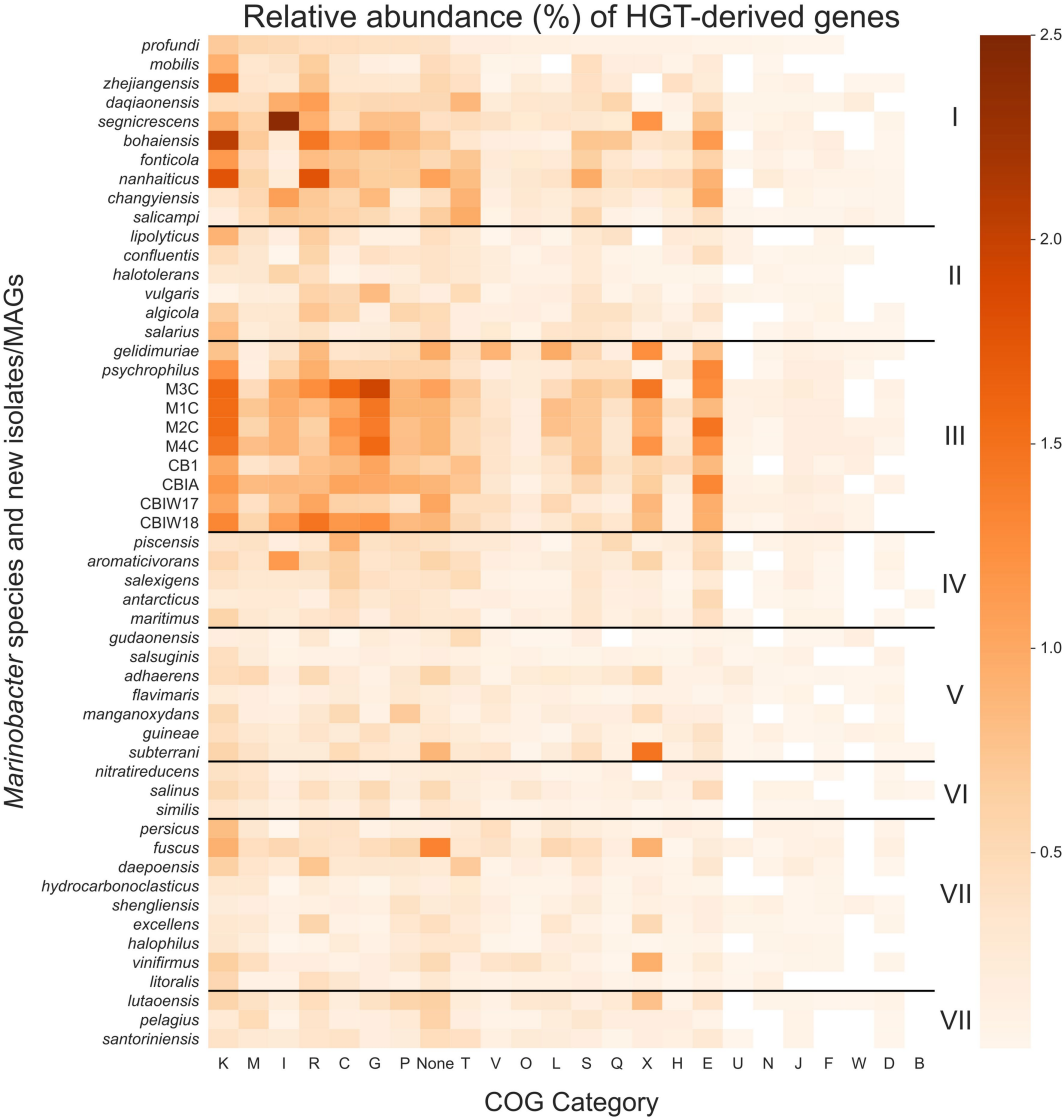
Heatmap of the relative abundance of genes derived by horizontal gene transfer (HGT) in each *Marinobacter* genome. Each row represents a single *Marinobacter* genome, and each column represents a COG category. Normalization was done by counting the number of potentially HGT-derived genes, dividing by the total number of genes in a genome, and converting to a percentage. The colormap ranges from 0% (lightest orange) to 2.5% (darkest orange). Black horizontal lines separate the heatmap into clades, with clade numbers I–VIII listed between the heatmap and scale bar. COG categories are defined as follows: K for transcription; M, cell wall/membrane/envelope biogenesis; I, lipid transport and metabolism; R, general function prediction only; C, energy production and conversion; G, carbohydrate transport and metabolism; P, inorganic ion transport and metabolism; T, signal transduction mechanisms; V, defense mechanisms; O, posttranslational modification, protein turnover, chaperones; L, replication, recombination and repair; S, function unknown; Q, secondary metabolites biosynthesis, transport and catabolism; X, mobilome: prophages, transposons; H, coenzyme transport and metabolism; E, amino acid transport and metabolism; U, intracellular trafficking, secretion, and vesicular transport; N, cell motility; J, translation, ribosomal structure and biogenesis; F, nucleotide transport and metabolism; W, extracellular structures; D, cell cycle control, cell division, chromosome partitioning; B, chromatin structure and dynamics; and None, no COG annotation available.

We searched each gene in each genome for signs of HGT using HGTector (Zhu et al., 2014), which employs a statistical approach to estimate the likelihood of a gene being derived from a close or distant relative that would indicate its acquisition *via* HGT. The patterns of HGT are of particular interest to life in cryopeg brines given their biogeographical isolation and continuous exposure to selective and stressful conditions. We calculated the proportion of genes in each genome (percentage of total genes) that were scored as putative HGTs (Figure 5; see Supplementary Data Sheet) and used this information along with NCBI COG annotations to score total proportion of HGT for each COG category in each genome. The mean proportion of total genes derived from HGT for the entire pangenome is 6.9 (±3.7)%, with a median of 5.3%, minimum of 2.5%, and maximum of 16.4%. Clade III alone, however, contained a mean of 12.5 (±2.9)% total genes derived from HGT; all other clades had lower means, in descending order: 8.8 (±2.9)% for Clade I, 5.5 (±2.9)% for Clade IV, 4.9 (±2.9)% for Clade II, 4.8 (±2.9)% for Clade VIII, 4.5 (±2.9)% for Clade VII, 4.2 (±2.9)% for Clade V, and 3.6 (±2.9)% for Clade VI.

Compared to other clades, Clade III had notably high proportions of putative HGTs from COG categories for transcription (K), carbohydrate transport and metabolism (G), amino acid transport and metabolism (E), energy production and conversion (C), and the mobilome (X), particularly prophages and transposons. These categories each contribute a mean proportion of total genes between 0.9% and 1.3% (Figure 5). HGT-derived genes without a COG annotation comprise a mean proportion of 0.9% of total genes for Clade III genomes. The most abundant HGT-derived genes found in the new cryopeg genomes were components of the TRAP-type C4-dicarboxylate transport system (COG1593, COG1638, and COG3090; category G), LysR family DNA-binding transcriptional regulators (COG0583; category K), and transposases (COG2801 and COG3039; category X). For other Clade III genomes, the most abundant HGTs found in the *M. gelidimuriae* genome were transposases (COG1943; category X), 5-methylcytosine-specific restriction endonuclease McrA (COG1403; category V), and site-specific recombinase XerD (COG4974; category L), while the most abundant HGTs found in the *M. psychrophilus* genome were DNA-binding transcriptional regulators from the LysR and GntR families (COG0583 and COG1802; category K), an ABC-type amino acid transport system (COG0765; category E), and components of the TRAP-type C4-dicarboxylate transport system (COG1593, COG1638, and COG3090; category G).

## Discussion

### The *Marinobacter* Pangenome

In this study, we used pangenomics as a tool to explore the general diversity of *Marinobacter* spp. and to contextualize within that known diversity a new set of genomes we obtained from subzero cryopeg brines. Pangenomic analyses can vary depending on the data used, such that a separate study of the *Marinobacter* pangenome using even more data and complete genomes may provide slightly different or more refined insights; we also acknowledge that variations in gene frequencies can differ between ecologically distinct populations of the same species (Cordero and Polz, 2014). Nevertheless, our analysis of the *Marinobacter* pangenome, generated from publicly available representative genomes of *Marinobacter* species and complete genomes from novel isolates and MAGs from the extreme cryopeg brine environment, clearly shows the immense diversity of genes encompassed by the members of this genus (Figure 3).

Among the 53 genomes analyzed here, the majority of the 24,580 detected gene clusters occurred as singletons (52% present in only one genome), with an additional 30% of gene clusters that could only be detected in 2–9 genomes, illustrating the tremendous diversity of genes in the *Marinobacter* pangenome as it currently exists. While the majority of singleton genes are of unknown function, genes related to the mobilome are more abundant among the singleton genes than in the core, where they are negligible, and thus reflect the role of genome modification processes that allow each genome to contain a unique set of genes. We approximated the core region of the pangenome to include gene clusters that are found in 50–53 of the genomes to avoid bias against core genes that may not be complete in the MAGs. This approximated core, which includes genes involved in common metabolic processes and genomic utility, contained <6% (1,465 gene clusters) of the total gene clusters. By comparison, a study of the pangenome of *Prochlorococcus*, based on the same methodology, indicated that this genus of cyanobacteria has a diverse pangenome with singletons and core constituting approximately 30% and 10% of all gene clusters, respectively (Delmont and Eren, 2018). The pangenome of *Pseudoalteromonas*, a Gammaproteobacterial relative of *Marinobacter*, has also been surveyed (albeit with different methodology) and found to be open, with singletons and core genes representing 80% and 7%, respectively, of all gene orthologs (Bosi et al., 2017). Both the *Prochlorococcus* and *Pseudoalteromonas* pangenomes are discussed as having a distribution of genes that correlates to the widespread success of their members in occupying extensive environments in the ocean. Given an even greater pangenomic diversity for *Marinobacter* than observed for *Prochlorococcus* and some similarity to *Pseudoalteromonas*, our findings highlight the ecological success demonstrated by the open structure of the *Marinobacter* pangenome.

### Biogeography and Phylogeny of *Marinobacter*

Plotting the isolation locations of the *Marinobacter* species discussed here (Figure 1; Supplementary Table 1) reveals that they are distributed globally and derived primarily from coastal marine environments. Though these locations represent a subset of the environments where *Marinobacter* spp. have been detected by other approaches, they allow us to make qualitative observations about the differences between species based on their origins. A few exceptions to the coastal marine setting include *M. halophilus* (Zhong et al., 2015) and *M. persicus* (Bagheri et al., 2013) isolated from terrestrial hypersaline lakes in China and Iran, respectively, and *M. subterrani* (Bonis and Gralnick, 2015) isolated from iron-rich saline fluids below the continental surface in Soudan Mine, Minnesota, United States. Species of *Marinobacter* have also been isolated from anthropogenic nearshore, saline environments, including oil-polluted wastewater, the source of *M. gudaonensis* (Gu et al., 2007) and *M. shengliensis* (Luo et al., 2015); wine-barrel-decalcification wastewater, the source of *M. vinifirmus* (Liebgott et al., 2006); and salted anchovies, which yielded *M. piscensis* (Hedi et al., 2015). The environments of isolation also generally correspond to their clade assignments in this study (Supplementary Table 1), though some associations are clearer than others. Clades I and IV contain isolates primarily from marine sediments; Clade II, from salterns and estuaries; Clade III, from subzero brines; Clades V and VIII, from a mix of deep and hydrothermal and seawater environments; and Clades VI and VII, from seawater (Figure 1; Supplementary Table 1). Exceptions to these descriptions of clade environments exist (e.g., pollution- and protist-associated isolates are dispersed throughout), but generally, *Marinobacter* spp. appear to occupy the niche for opportunistic heterotrophs in nutrient-rich saline environments across the globe.

The phylogenetic tree based on a concatenation of a subset of core genes from the pangenome (Figure 2B) represents a comprehensive genomic perspective for assessing genome relatedness in the pangenome. In using this method to assign the genomes into clades, we were able to recognize patterns of species distributions by environment or common genomic characteristics. In this core tree (Figure 2B), the novel cryopeg genomes clustered together in Clade III with the two other genomes from subzero brine environments (Figure 1): *Marinobacter psychrophilus*, isolated from sea ice in the Canadian Basin of the Arctic Ocean (Zhang et al., 2008), and *M. gelidimuriae*, isolated from subglacial brine flowing from Blood Falls in Antarctica (Chua et al., 2018). This initial grouping of the novel genomes along with other *Marinobacter* genomes from subzero brines of both polar regions suggests that environmental setting plays a role in the differentiation of this genus.

The traditional rooted universal phylogenetic tree (Figure 2A), constructed to validate organization of the core tree (Figure 2B), upheld the clustering of subzero brine *Marinobacter* genomes in Clade III. It also showed that this group branches from the rest of the genus earlier than any other clade. Within Clade III, the MAGs form their own early branch on the rooted universal tree (Figure 2A), though this feature may be an artifact of their reconstruction from natural samples that contain a greater sequence diversity from the *in situ* population than the genomes from clonal isolates. In this tree, except for Clades I and II, the other clades mostly remained intact, although neighboring clade organization differed between the trees. A third tree based on ANI scores (Figure 2C) also supports the distinct clustering and separate branching of Clade III from the rest of the genus. The support of Clade III by these multiple methods indicates that this branch of subzero brine-dwelling *Marinobacter* spp. evolved from a common lineage. Cryopeg brines and other subzero brine ecosystems are well known to be characterized by low biodiversity (Mikucki and Priscu, 2007; Niederberger et al., 2010; Boetius et al., 2015; Cooper et al., 2019), a signature of the highly selective nature of these environments. The most parsimonious explanation of the relation of Clade III species, given the low biodiversity of the environments they inhabit, their high *in situ* abundance, and their consistent clustering using multiple methods, is that they have successfully and independently occupied this niche through competitive growth and survival under extreme conditions over long periods of time.

The consistent clustering of Clade III genomes, along with their shared origins in subzero brines from both polar regions (Figure 1), raises questions about the relatedness of this clade to the rest of the genus. Using ANI (Supplementary Data Sheet), we observed that the new genomes from cryopegs are all highly related (>99%), though the MAGs might have differed with higher completion. The closest relatives to the cryopeg genomes are *M. gelidimuriae* and *M. psychrophilus* (also in Clade III) with ANI scores of approximately 93.1% and 90.7%, respectively. A threshold of 95%–96% has been suggested as a cutoff for species delineation by ANI, along with a cutoff of 98.65% similarity of the 16S rRNA gene (Kim et al., 2014). *Marinobacter gelidimuriae* and *M. psychrophilus* have alignment coverage values of 61.4% and 71.3% with the cryopeg genomes, which are notably higher than values for the rest of the *Marinobacter* genomes to Clade III genomes (Supplementary Data Sheet). *Marinobacter gelidimuriae* and *M. psychrophilus* also share 99.3% and 99.0% 16S rRNA gene similarity with the novel cryopeg genomes, respectively. Though the 16S rRNA gene similarity is high relative to both of these species, *M. gelidimuriae* and *M. psychrophilus* themselves are sufficiently distinct by ANI and 16S rRNA gene similarity (Chua et al., 2018) to delineate them as separate species. Given that the ANI scores of the new cryopeg isolates fall below the defined cutoff and their alignment coverage to the closest relative species is low, we suggest that these new isolates represent, at a minimum, a novel species.

As a group, Clade III *Marinobacter* genomes have much higher ANI with each other than with other *Marinobacter* species; i.e., ≥90.6% ANI between Clade III members versus 72.7%–76.4% ANI between Clade III members and other clades (Figure 3; Supplementary Data Sheet). Along with low ANI between *Marinobacter* spp. (median of approximately 75%), we also observed generally low alignment coverage values between all species, with the majority ranging between approximately 30% and 50% (Supplementary Figure S1). The variability in ANI and alignment coverage observed here for the *Marinobacter* genus falls within the variability discussed for defining genus demarcation (Barco et al., 2020). However, the early branching of clade III in the rooted phylogenetic tree (Figure 2A), along with the high level of ANI between Clade III species, lower levels of ANI between Clade III and all other species (Figures 2C, 3), and significantly lower GC content for Clade III than all other clades (*p* = 3 × 10^−6^), calls into question whether this clade truly belongs within the genus *Marinobacter*. Clade III may represent a new genus of Bacteria, related to but distinct from *Marinobacter*.

### Metabolic Potential of the *Marinobacter* Pangenome

Previous studies have explored the physiological diversity of cultured species of *Marinobacter* and demonstrated the benefits of metabolic versatility to evolutionary success in saline environments (Handley and Lloyd, 2013). The genus is well known for its ability to metabolize a large variety of hydrocarbons, carbohydrates, and amino acids as sources of carbon (Gauthier et al., 1992; Green et al., 2006; Liu et al., 2012; Handley and Lloyd, 2013). Few of the species are considered strict aerobes, but several are facultatively anaerobic, with an ability to use alternative electron acceptors, such as nitrate or fumarate (Takai et al., 2005), and to use arsenic, iron, and manganese for redox cycling (Handley et al., 2009; Wang et al., 2012). In this study, we explored the functional potential of the genus using KEGG annotations of gene clusters in the pangenome.

After annotating each gene in each genome with KOs and processing further to determine which metabolisms were available for each species of *Marinobacter* (Figure 4; Supplementary Data Sheet), we found that the glycolysis pathway was not complete for any species of *Marinobacter*. Rather, the Entner–Doudoroff pathway, an alternative to glycolysis, was complete in all but one of the genomes (*M. gelidimuriae*). The Entner–Doudoroff pathway has been discussed as an adaptive alternative to glycolysis, particularly for psychrophilic bacteria as exemplified by *Colwellia psychrerythraea* 34H (Czajka et al., 2018), because it requires less total protein mass and fewer temperature-constrained enzymatic reactions to convert glucose to pyruvate, even though it nets one less ATP (Conway, 1992). Additionally, each genome encodes the glyoxylate shunt pathway, which is an alternative to the tricarboxylic acid (TCA) cycle, using five of the eight enzymes in the TCA cycle and two unique enzymes (net one less enzyme). The glyoxylate shunt bypasses the carbon dioxide-producing steps of the TCA cycle and has been hypothesized to play a role in oxidative stress response. It is also essential for acetate and fatty acid metabolism in bacteria (Ahn et al., 2016), both of which have been observed as organic substrates for *Marinobacter* (Handley and Lloyd, 2013). The presence in all genomes of complete metabolic pathways for each of the 20 common amino acids (with the exception of asparagine metabolism, for which some genes are missing in 12 of the genomes) suggests that *Marinobacter* has a competitive advantage over other heterotrophic psychrophiles with more limited metabolisms to meet their amino acid requirements. As most *Marinobacter* species also encode flagella and chemotactic pathways, they appear to have the capacity to actively seek such labile organic resources, which could occur even under subzero hypersaline conditions, as demonstrated for *C. psychrerythraea* 34H (Showalter and Deming, 2018). The genetic potential to seek resources and utilize a broad range of organic compounds is clear indicators of the diverse and opportunistic lifestyles for which *Marinobacter* spp. are known, while the discovery of streamlined metabolic pathways despite lower energetic yield highlights the metabolic tradeoffs for survival in an extreme environment.

### Distinct Characteristics of the Subzero Brine Clade

Clade III genomes encoded several KEGG pathways that were less well represented in the rest of the pangenome and may indicate adaptation to their extreme environment (Figure 4). Among these are nitrogen and sulfur oxidation and reduction pathways, metal transport pathways, and Type I secretion systems. The availability of this variety of redox reactions adds support to previous observations of the facultatively anaerobic lifestyle of *Marinobacter* (Takai et al., 2005; Handley et al., 2009; Handley and Lloyd, 2013; Chua et al., 2018) and fits with models of redox potential available in anoxic marine sediments (Zobell, 1946; Lam et al., 2019), which are likely the source material for cryopegs before incorporation into permafrost (Iwahana et al., 2021). Clade III *Marinobacter* spp. also appear well equipped for ion and metal transport across the membrane, befitting the concentrated ionic and likely anoxic or hypoxic environments of subzero brines. Transport mechanisms are particularly important in a brine setting where osmolarity and metal toxicity need to be closely monitored and regulated by the cell for survival (Csonka, 1989; Firth et al., 2016). In addition to the economical utility of the Entner–Doudoroff and glyoxylate shunt pathways available to the genus, Clade III genomes (except for *M. gelidimuriae*) also encode all of the components necessary for the C-P lyase system which allows for the foraging of phosphorous from organophosphates when phosphorous may not be readily available in the biologically preferred inorganic form (Kamat and Raushel, 2013; Stosiek et al., 2020). The species in Clade III were each isolated from subzero, hypersaline environments where redox availability and membrane regulation are critical to survival and competition (Zhang et al., 2008; Chua et al., 2018; Cooper et al., 2019). The metabolic characteristics encoded in their genomes thus reflect the flexible and adaptive strategies employed for their success in subzero brines.

An analysis of the COG annotations revealed several functions that appear to be enriched in Clade III compared to the other clades (Supplementary Data Sheet), including membrane transport systems, compatible solute metabolism, and genetic regulatory mechanisms. ABC-type membrane transport systems, the most enriched functions in Clade III, likely enable the essential transport of organic substrates, ions, and compatible solutes (Schneider and Hunke, 1998). The presence of bacteriorhodopsin in Clade III, along with the KEGG pathway for retinal biosynthesis (Figure 4), indicates that transmembrane pigments may also play a role in the fitness of these species. This functional capability is only observed in the isolates from cryopeg brines and in their closest relative, *M. psychrophilus*, isolated from sea ice. Bacteriorhodopsin is known as a transmembrane proton-pumping protein that uses light energy to catalyze the process (Lanyi, 2004). The presence of this gene in the cryopeg genomes is enigmatic, as these species have been isolated from surface radiation for thousands of years (Iwahana et al., 2021). We hypothesize that this protein, if expressed, may play a role in ion exchange across the membrane (Culligan et al., 2014; Oren, 2015), assisting in salt tolerance in the hypersaline setting of the cryopeg. As the cryopeg ecosystem is under continuous osmotic pressure, more than one means to regulate ion exchange may increase fitness. The production of compatible solutes, such as glycine betaine, is an essential means for maintaining cellular integrity. In cryopeg genomes, the enrichment of enzymes involved in the metabolism of sarcosine to glycine appears to reflect the importance of compatible solute production in a subzero brine habitat, as this metabolism has also been documented in sea-ice bacteria (Collins and Deming, 2013; Firth et al., 2016).

In addition to functions that would modulate transmembrane transport and osmolarity under extreme conditions, we found an enrichment of genes in Clade III related to genetic regulation and modification. These genes may have particular evolutionary importance for bacteria in cryopeg brines where continuously low temperatures mean long generation times (Mykytczuk et al., 2013; Chua et al., 2018). To combat environmental stressors while managing slow rates of vertical evolution, we hypothesized that *Marinobacter* may need to employ more regulatory and recombinant strategies than may be typical to maintain a competitive state. Stress response proteins, which are transcriptional regulators that can be related to salt and cold stress (Starosta et al., 2014), were enriched in Clade III; they may play a role in managing gene expression under the extreme conditions of a subzero brine. A recombination-promoting DNA endonuclease and a plasmid stabilization module were also found to be enriched in Clade III. These mobilome genes may play a role in maintaining genetic diversity and fluidity in the population (Toussaint and Chandler, 2012). Though enrichment in a clade does not confirm that traits are present specifically for their adaptive capacity to a given environment, we consider the potential of enriched mobilome genes in Clade III important to developing a more nuanced understanding of adaptive success in the subzero brine environment.

### HGT Signatures Across the *Marinobacter* Pangenome

Due to the extreme temperature and salinity conditions of subzero brines, bacterial growth rates in these environments are expected to be slow, yet the concentrating effect of freezing saline water means that infection pressure from viruses within the resulting brines may be much higher than in their source waters (Wells and Deming, 2006; Collins and Deming, 2011). In subzero cryopeg brines with high bacterial densities, cell–cell interactions are also expected to be high (Rapp et al., 2021). Given the anticipated low rates of vertical inheritance associated with slow growth rates, we hypothesized that HGT plays an essential role in the evolution and adaptation of bacteria in the extreme subzero hypersaline environment. Our hypothesis corresponds with other studies that indicate the importance of HGT in extreme environments (Collins and Deming, 2013; Feng et al., 2014; Li et al., 2014; Raiger Iustman et al., 2015; Bosi et al., 2017), adding to the collective understanding of the role of HGT in evolution. We measured putative HGT events in each *Marinobacter* genome using an alignment-based approach that identifies genes that are more closely related to distant taxonomic relatives than to close relatives (Zhu et al., 2014). This method reveals genes that may have been transferred to a genome from another genome outside of its own taxonomic family (for the analytical settings used in this study). We observed an overall higher abundance of HGT genes in Clade III genomes relative to other clades and found that these genes belong to categories that may be involved in conferring adaptation to subzero brine conditions (Figure 5).

Among the most abundant HGT genes were those in the COG categories for transcription (K) and the mobilome: prophages, transposons (X). These transferred genes may play roles in genome flexibility under extreme conditions or simply be signatures of the mechanisms of HGT. Category K includes stress response transcription factors, such as cold and salt stress response proteins (Graumann et al., 1997; Starosta et al., 2014), which would contribute to the adaptive preparedness displayed by *Marinobacter* spp. in Clade III. Category X includes a variety of genes, from transposases, integrases, and recombinases to phage-associated genes, each of which may play a role in genome modification or HGT (Toussaint and Chandler, 2012). We cannot disentangle the possibility of virally transduced or conjugation-based HGT from these data, but both are strong possibilities in the densely populated settings of subzero brines (Collins and Deming, 2011, 2013; Rapp et al., 2021). The cryopeg brines that yielded the new *Marinobacter* isolates and MAGs contained high densities of both bacteria and virus-like particles (approximately 10^8^ ml^−1^ each; Cooper et al., 2019), and our previous work on the same subzero brine system showed that virally mediated gene transfer can directly affect microbial fitness (Zhong et al., 2020).

We also identified notable abundances of HGT events from COG categories for carbohydrate transport and metabolism (G), amino acid transport and metabolism (E), and energy production and conversion (C) in Clade III genomes. As these processes reflect the metabolic flexibility displayed by the genus *Marinobacter* as a whole (Handley and Lloyd, 2013), the abundance of HGT-derived genes in these categories and potentially those of unknown function highlights the importance of flexible metabolic potential in an extreme environment (Collins and Deming, 2013; Fuchsman et al., 2017). The cryopeg ecosystem is isolated from the surface environment, leaving bacteria to survive on the nutrients available *in situ*. Over a long timeframe, many of these nutrients may derive from cryptic growth, where cytoplasmic contents released upon cell lysis (due to viral lysis or other forms of cell death) are recycled in support of a persistent, slow-growing community (as presented by Rapp et al., 2021). As labile organics are consumed, more recalcitrant (sediment-adsorbed or permafrost-derived) forms of organic substrates may become important sources of nutrients for the community. Extracellular enzymes that degrade organic matter have been demonstrated to be active under cryopeg conditions (Showalter and Deming, 2021), and the *Marinobacter* species characterized here are well equipped genetically to take advantage of the variety of substrates that can be produced by this general process. We suggest that *Marinobacter* spp. in subzero brines have succeeded at competitive opportunism by maintaining and expanding broad genomic potential for the consumption of a variety of metabolites using multiple redox sources (electron acceptors). The combination of genetic and metabolic flexibility potentially achieved through HGT highlights the key strategies employed by this genus that has allowed it to occupy its competitive position globally in saline environments and endemically in subzero brines.

## Conclusion

Pangenomics provides a unique approach to explore the evolutionary history and ecological context of microorganisms. The pangenome of *Marinobacter*, a globally distributed genus of bacteria inhabiting saline environments, displays genetic versatility that complements previous physiological studies. There is a tremendous amount of rare (variable) gene content in this pangenome, and a relatively small, conserved (core) region. This pattern of gene distribution reflects the metabolically flexible lifestyle across a wide range of saline environments for which the genus is known. Here, we have produced a set of genomes belonging to a novel species of *Marinobacter*, successfully cultivated from cryopeg brines and recovered as high-quality MAGs, that represents the dominant taxon in the sampled communities. This species is most closely related to other *Marinobacter* species found in sea ice and in subglacial brines from both Arctic and Antarctic settings; together they represent a unique clade of *Marinobacter*. Members of this subzero brine clade share genetic features thought to be important in adapting to extremely cold and hypersaline environments. Patterns of phylogeny and ANI indicate that this clade may have diverged early from the genus *Marinobacter* and may even represent a novel genus of bacteria endemic to subzero brine environments, which will be explored in future analyses. The genetic inventory of this clade alongside ecological context gives a clear depiction of the competitive and selective forces that drive microbial evolution in these extreme environments, including metabolic versatility, broad membrane transport and environmental sensory functions, and genomic flexibility *via* the mobilome and horizontal gene transfer.

## Data Availability Statement

The datasets presented in this study can be found in online repositories. The names of the repository/repositories and accession number(s) can be found in the article/Supplementary Material. The newly generated data are all included in NCBI BioProject PRJNA540708.

## Author Contributions

ZC and JD conceived the study. ZC and AS isolated the new strains. Z-PZ assembled the polished long-read metagenomes. ZC performed all of the data analyses, with support of interpretations from JR, RA, and JD and wrote the first draft of the manuscript, with critical input from all authors during revision. All authors contributed to the article and approved the submitted version.

## Funding

This research was supported by the Gordon and Betty Moore Foundation grant no. 5488 to JD, with high-performance computing resources provided by the laboratory of RA. Illumina sequencing services were provided by the U.S. Department of Energy Joint Genome Institute, a DOE Office of Science User Facility, which is supported by the Office of Science of the U.S. Department of Energy under contract no. DE-AC02-772 05CH11231.

## Conflict of Interest

The authors declare that the research was conducted in the absence of any commercial or financial relationships that could be construed as a potential conflict of interest.

## Publisher’s Note

All claims expressed in this article are solely those of the authors and do not necessarily represent those of their affiliated organizations, or those of the publisher, the editors and the reviewers. Any product that may be evaluated in this article, or claim that may be made by its manufacturer, is not guaranteed or endorsed by the publisher.

## Supplementary Material

Supplementary Figure S1 Histogram of average nucleotide identity (ANI) and alignment coverage values calculated pairwise for each genome in the *Marinobacter* pangenome. The *x*-axis displays the proportional values of identity and alignment coverage, respectively. The *y*-axis displays the count of observations.
