# Supplementary figures and images for: Evolutionary Divergence of Marinobacter Strains in Cryopeg Brines as Revealed by Pangenomics
Source: Front Microbiol. 2022 Jun 6;13:879116. doi: 10.3389/fmicb.2022.879116 (PMC9207381; doi:10.3389/fmicb.2022.879116)

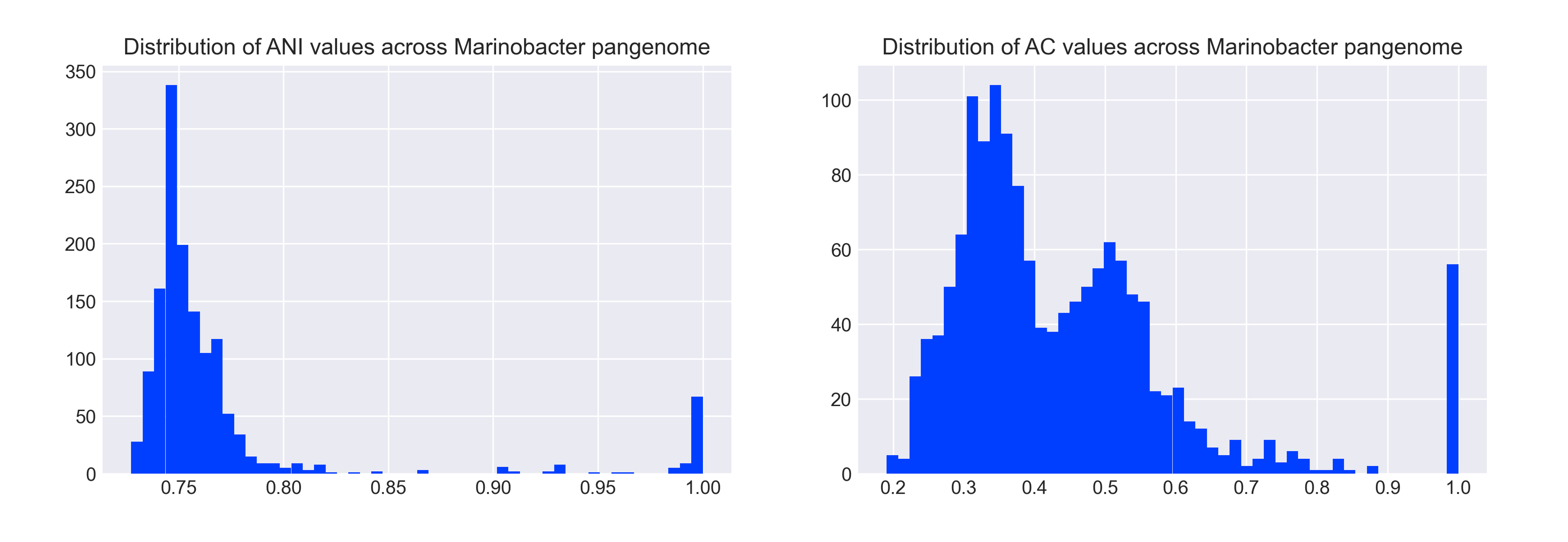

Supplement: Supplementary Figure S1 — Histogram of average nucleotide identity (ANI) and alignment coverage values calculated pairwise for each genome in the Marinobacter pangenome. The x-axis displays the proportional values of identity and alignment coverage, respectively. The y-axis displays the count of observations. [file Image_1.JPEG]
